# Analysis of ischemic stroke-mediated effects on blood–brain barrier properties along the arteriovenous axis assessed by intravital two-photon imaging

**DOI:** 10.1186/s12987-024-00537-5

**Published:** 2024-04-15

**Authors:** Jil Protzmann, Felix Jung, Lars Jakobsson, Linda Fredriksson

**Affiliations:** 1 Department of Medical Biochemistry and Biophysics, Division of Vascular Biology, Karolinska Institutet, Solnavägen 9, Stockholm, Sweden 17165; 2 Department of Neuroscience , Karolinska Institutet, Solnavägen 9, Stockholm, Sweden 17165

**Keywords:** Blood–brain barrier breach, Two-photon microscopy, Ischemic stroke, Arteriovenous axis, Vascular remodeling, Longitudinal imaging, Intravital imaging, Leakage

## Abstract

Early breach of the blood–brain barrier (BBB) and consequently extravasation of blood-borne substances into the brain parenchyma is a common hallmark of ischemic stroke. Although BBB breakdown is associated with an increased risk of cerebral hemorrhage and poor clinical prognosis, the cause and mechanism of this process are largely unknown. The aim of this study was to establish an imaging and analysis protocol which enables investigation of the dynamics of BBB breach in relation to hemodynamic properties along the arteriovenous axis. Using longitudinal intravital two-photon imaging following photothrombotic induction of ischemic stroke through a cranial window, we were able to study the response of the cerebral vasculature to ischemia, from the early critical hours to the days/weeks after the infarct. We demonstrate that disruption of the BBB and hemodynamic parameters, including perturbed blood flow, can be studied at single-vessel resolution in the three-dimensional space as early as 30 min after vessel occlusion. Further, we show that this protocol permits longitudinal studies on the response of individual blood vessels to ischemia over time, thus enabling detection of (maladaptive) vascular remodeling such as intussusception, angiogenic sprouting and entanglement of vessel networks. Taken together, this in vivo two-photon imaging and analysis protocol will be useful in future studies investigating the molecular and cellular mechanisms, and the spatial contribution, of BBB breach to disease progression which might ultimately aid the development of new and more precise treatment strategies for ischemic stroke.

**Supplementary Information:**

The online version contains supplementary material available at 10.1186/s12987-024-00537-5.

## Background

Stroke is a leading cause of death and disability worldwide, with over 12 million people suffering from a stroke every year [10]. 85% of stroke patients experience an ischemic stroke, which is characterized by the blockage of a cerebral blood vessel leading to strongly reduced regional cerebral blood flow and consequently hypoxia and hypoglycemia in the affected brain area. If blood flow is not reestablished within a few hours after the infarct, neuronal cell loss and permanent functional impairment arise which can entail the need for costly, life-long medical care of the patient [30].

A hallmark of ischemic stroke is blood–brain barrier (BBB) disruption and dysfunction [28, 43], which cause extravasation of blood-borne molecules and infiltration of cells from the vessel lumen into the brain parenchyma and greatly exacerbate stroke pathology [26]. In humans, early BBB breach has been reported to be an independent predictor for hemorrhagic transformation and poor clinical outcome [19]. Despite the pivotal role of BBB breakdown in ischemic stroke progression our understanding of the underlying dynamic processes remains limited.

Preclinical BBB research has previously relied on static, ex vivo assessment of disease parameters including BBB breach and immune cell infiltration [12, 33, 34]. This required large cohorts of animals and did not accommodate studies on dynamic changes, nor correlation between acute (e.g., extent of BBB breakdown) and chronic (e.g., functional recovery) parameters, within the same individual. Advances in intravital imaging techniques, including magnetic resonance imaging (MRI) and two-photon microscopy, now offer ways to monitor stroke progression in real-time in the living animal [5]. While MRI is non-invasive and can detect stroke lesion volume, edema, and hemorrhages brain-wide [25, 40], it lacks the resolution to study contribution to stroke progression at the cellular level. In contrast, two-photon microscopy offers the spatial and temporal resolution to longitudinally study stroke progression in real-time at (sub-)cellular resolution [21, 29] and has been applied in stroke research to investigate microglia activation [14], collateral blood flow via leptomeningeal anastomoses [23] as well as mechanisms involved in post-stroke vascular plasticity [41, 42]. In addition, in vivo two-photon microscopy has been used to study trans- and paracellular mechanisms of BBB breakdown 6 hours post occlusion in a filament model of ischemic stroke [17]. Clinically, however, the first few hours are crucial as clinical prognosis and treatment options depend on how much time has elapsed from stroke onset, thus highlighting the need for studies within this early time window. Here, we have established an in vivo two-photon imaging and analysis protocol allowing longitudinal studies of BBB breakdown commencing as early as 30 min after induction of large artery photothrombotic occlusion in mice. We showed that stroke-induced BBB breach can be studied at single vessel resolution and developed an analysis method to quantify vascular leakage and perfused vessel density in the three-dimensional (3D) space. Using endothelial cell reporter mice, we demonstrate that hemodynamic parameters and BBB breach can be followed along the arteriovenous (AV) axis, and we detected vessel type-specific vulnerabilities to the ischemic insult. This is of interest since cells in the neurovascular unit, which establish the BBB, show both transcriptional and phenotypic differences along the AV axis likely reflecting functional specifications [37]. Thus, this imaging protocol will be a useful tool to investigate how AV zonation influences BBB properties and vascular function in the acute phase after ischemic stroke and how this correlates to subacute/chronic injury responses and functional recovery. Ultimately, our experimental setup can be used to delineate what vascular segments to target aiming to improve cerebrovascular function after stroke, the mechanism of such strategies and when they might be most effective.

## Material and methods

### Ethical statement

All experiments in this study were approved and performed in accordance with the guidelines from the Swedish National Board for Laboratory Animals and the European Community Council Directive (86/609/EEC). The ethical permits N236-15 and 14960–2020 were approved by the North Stockholm Animal Ethics Committee. Animal welfare guidelines, as subsumed by the 3R principles, were followed at any time and animals were closely monitored by the researchers, technical staff and veterinarian team of Comparative Medicine, Karolinska Institute, throughout the progress of the experiments.

### Mouse models

Adult (2–9 months old), male and female C57BL/6 J mice (The Jackson Laboratory) and Cldn5(BAC)-GFP mice backcrossed to the C57BL/6 J background (kindly provided by Christer Betsholtz Lab, Uppsala University and within referred to as ‘Claudin5-GFP’) were used in this study. Claudin5-GFP mice express cytosolic GFP under the control of the Claudin5 promoter [37, 38]. A complete list of mice used in this study can be found in Additional file 1: Table S1. The mice were housed in groups until the beginning of the experiment and afterwards housed individually or in pairs in individually ventilated cages (GM500, Scanbur) at a 12 h dark/light cycle at a constant temperature of 22 °C with 50% relative humidity. Mice had access to water and chow ad libitum*,* and cage enrichment was provided.

### Cranial window implantation

Cranial window implantations were performed as previously described [15, 16]. Briefly, the mouse was deeply anesthetized with isoflurane, placed on a heating pad and head-fixed in a rotatable surgical frame (Narishige SGM-4 with Anesthetic Mask, MicroSystem). The eyes were covered with eye ointment (Viscotears). The mouse received 5 mg/kg carprofen (Rimadyl bovis vet., Zoetis) and 0.06 mg/kg buprenorphine (Temgesic, Indivior UK Limited) for pain treatment as well as 2 mg/kg dexamethasone (Dexadreson vet., MSD animal health) to reduce brain swelling and inflammation (all subcutaneously; s.c.). The fur was removed using hair removal cream (Veet) at the site of surgery and local analgesic lidocaine (Lidocaine Accord, Accord) was injected (s.c.). The skin was disinfected with iodine (Jodopax vet., Pharmaxim AB), before removing a flap of skin over the left hemisphere of the skull between the left ear and eye. The gelatinous periosteum was removed, and the surface of the skull was roughened with a drill for increased bonding between bone and glue. A small metal bar (split headpost small, Luigs & Neumann) was attached to the skull of the left hemisphere with super glue (Loctite, Henkel). The cortical branch of the middle cerebral artery (M2CA) was made accessible by detaching the temporal muscle from the skull. After visualizing the superficial cerebral blood vessels by moistening the skull with phosphate buffered saline (PBS), a small craniotomy of 3 mm was performed above the M2CA. When removing the bone, attention was paid to leaving the dura mater intact. A glass coverslip (3 mm diameter, #0, Multi Channel Systems GmbH) was placed into the opening and sealed with dental cement powder (Paladur) mixed in super glue. The surrounding tissue and metal bar were generously covered with dental cement in order to seal off the wound. The mouse was allowed to wake up in a heated cage and received 0.06 mg/kg buprenorphine and 2 mg/kg dexamethasone (both, s.c.) 24 h after surgery (additional doses were given 48 h and 72 h if needed). The mouse recovered for a minimum of 14 days before starting the imaging protocol to allow the glial reaction to cease [13].

### M2CA occlusion through the cranial window

The protocol for photothrombotic stroke induction was adapted from Su et al. [34]. The mouse was deeply anesthetized with isoflurane, placed on a heating pad and head-fixed in a surgical frame. The eyes were covered with eye ointment and the mouse received 40 mg/kg of the photoactivatable dye rose bengal (Fisher Scientific) diluted in PBS retroorbitally (r.o.). Stable occlusion of the M2CA (M2CAO) was induced through the cranial window by pointing a 3.5 mW green light laser (540 nm, Melles Griot) on the M2CA for 20 min. Relative tissue perfusion was measured with a laser Doppler flow probe (Type N (18 gauge), Transonic Systems) connected to a flowmeter (Transonic model BLF22) and total occlusion was assumed when the relative tissue perfusion had dropped to less than 30% of its original value. Mice were either allowed to wake up in a heated cage or directly transferred to the two-photon microscope. Sham control mice underwent the same surgical procedure, but PBS was administered instead of rose bengal.

### Two-photon imaging

In order to visualize blood flow in the living mouse, 100 µl of 10 mg/ml fluorescent tracer (FITC70 or TRITC70, TdB Labs) were intravenously (i.v.) injected via the lateral tail vein prior to imaging alternatively mixed in with rose bengal and administered r.o. on the day of stroke. The mice were imaged once 1–3 days before stroke induction (pre M2CAO), immediately after M2CAO (30–120 min post stroke) and at various time points thereafter (up to 7 d post stroke). During imaging, the mouse was anesthetized with isoflurane, placed on a heating pad and head-fixed under the microscope (TSC SP8 multiphoton system, Leica) with a 3D articulated arm (Luigs & Neumann). During the M2CAO imaging session, the animal remained inside the microscope under isoflurane anesthesia the entire time (up to 120 min). Eye ointment was applied. As the cranial window above the M2CA is located around 3–5 mm laterally from the sagittal suture, the head of the animal was tilted in order to position the cranial window perpendicularly to the objective lens. The cranial window was cleaned with 70% ethanol before every imaging session. A 25x/1.0 NA water immersion objective (motCORR VISIR, Leica) was used which was heated to 37 °C by an objective mantle heater (ALAOBJ heater and ALAHOT-1 temperature controller, Scientifica) as a lowering in brain temperature has been reported to slow down cerebral blood flow and thus affect oxygenation of the tissue [27]. Epifluorescent images of the superficial vasculature were acquired using a monochrome digital camera (DFC365 FX, Leica). For two-photon excitation a pulsed Ti:Sapphire infrared laser (Chameleon S, Coherent) was used at the wavelengths of 880 nm to simultaneously excite Claudin5-GFP and TRITC70, and 920 nm to excite FITC70. Signals emitted in the green and red spectrum were separated using a 560 nm long-pass filter (Leica). The FITC70 and Claudin5-GFP signal was filtered with a 525/50 nm band-pass filter (Semrock) and collected with a photo-multiplier tube (Leica). The TRITC70 signal was filtered with a 585/40 nm band-pass filter (Semrock) and collected with a hybrid detector (Leica). The LAS X software (Leica) was used for image acquisition. Z-stacks of 591 µm × 591 µm × 1 µm (xyz) covering 100–300 µm depth, at 1024 × 1024 pixel resolution (0.58 µm/pixel) were acquired. In each animal, stacks from alternating positions were recorded. Time lapses of 444 µm × 444 µm × 1.72 s (xyt) covering 7.5–28.5 min at 1024 × 1024 pixel resolution (0.43 µm/pixel) were acquired and, if needed, registered using the *moco* plugin in ImageJ/Fiji [9]. Video annotations were added using the *annotate_movie* plugin in ImageJ/Fiji [6].

### Measurement of stroke infarct volume

In order to assess infarct volume 72 h after stroke or sham surgery, mice were deeply anesthetized with isoflurane, decapitated and brains were collected. 2 mm thick coronal sections of the brains were cut using an acrylic mouse brain matrix (ALTO) and matrix cutting blades (ALTO). The sections were immediately transferred to freshly prepared 4% 2,3,5-triphenyltetrazolium chloride (TTC, Sigma Aldrich) in PBS for 20 min. TTC is a dye that is metabolized by mitochondrial enzymes of living cells into a red compound and thus discriminates between viable (red) and damaged/dead (white) tissue. Note that large nerve tracts (e.g., cranial nerves, corpus callosum, etc.) remain white after TTC staining, due to the high lipid content in the insulating myelin sheets, even when being metabolically active. TTC-stained sections were captured using a digital camera (PowerShot SX200 IS, Canon) attached to a dissection microscope (Leica). Lesion volumes were analyzed with ImageJ/Fiji using the following formula:

V_%stroke_ = (∑(infarct area)/∑(total area of ipsilateral hemisphere))*100, where V_%stroke_ is infarct volume calculated as percent of the ipsilateral hemisphere.

## Image analysis

### Vascular leakage and vessel density

Image analysis for vascular leakage and lengths of perfused vessels was based on a semi-automated tracing algorithm (Simple Neurite Tracer, SNT [22]) in ImageJ/Fiji. First, a Gaussian Blur filter (sigma = 1) was applied to the original z-stack in order to reduce noise and increase edge-detection. Semi-automatic vessel tracing was done by creating point-based vessel paths for each vessel in the dataset. Traced vessel paths were used as seed points for automatically filling the vessel lumen using the SNT algorithm. Extraluminal FITC70 signal was separated from the FITC70 signal in the vessel lumen by subtracting the traced vessels from the original z-stack using custom routines in Python. Note that due to the curvature of the brain, image planes close to the meninges contained brain parenchyma as well as subarachnoid space. The presence of pial capillaries enabled a crude segmentation of brain and subarachnoid space by manually traced regions-of-interest (Additional file 2: Fig S1). Vascular leakage of FITC70, including both parenchymal and subarachnoid leakage, was calculated by normalizing the extraluminal to the luminal signal thereby accounting for variation in imaging quality from day to day and for variations in fluorescent tracer injection that could arise from the two different application routes (r.o. administration on the day of M2CAO induction versus i.v. administration via the lateral tail vein during the consecutive days after M2CAO and when recording the baseline signal pre M2CAO). Due to noise in the acquired images and technical limitations when separating the luminal from the extraluminal signal on a single pixel level, the pre M2CAO time point does not equal zero.

### Classification of cerebral vessels

In reporter mice, cerebral blood vessels were manually classified pre M2CAO using the following criteria. Arteries (A) possess elongated, streamlined endothelial cells and are > 45 µm in diameter, while arterioles (Ae) branch off arteries and are 10–45 µm in diameter. Veins (V) have irregular, uneven endothelial cells and are > 50 µm in diameter, while venules (Ve) converge to form veins and are 10–50 µm in diameter. A single endothelial cell covers the whole lumen of a capillary (C) which has a diameter of < 10 µm.

### Local BBB leakage

To analyze local BBB leakage, parenchymal TRITC70 accumulation was manually attributed to the nearest vessel and the vessel diameter and vascular identity registered. If two vessels were equally close to a site of local leakage and clear attribution to either of the vessels was not possible, both were included in the analysis. The distance of TRITC70 spreading was measured from the vessel wall to the most distant continuous TRITC70 signal. Spreading distance was determined at 1–2 µm intervals along the length of the site of leakage and averaged.

### Vessel diameter

Vascular diameter in segments of arteries, arterioles, capillaries, venules, and veins was measured before (pre M2CAO) and after (post M2CAO) stroke induction. When a vessel branched it was counted as two new segments. The change in vessel diameter was assessed by normalizing the diameter post M2CAO to the diameter pre M2CAO.

## Statistical analysis

Data analysis was performed using the statistical software Prism 9 (GraphPad). For statistical analysis two-tailed *t*-tests (paired if applicable) were performed. Statistical significance was defined as *p ≤ 0.05, **p ≤ 0.01 and ***p ≤ 0.001. Data are presented as mean ± S.D., if not indicated otherwise.

## Results

### *Establishment of a model for high-resolution two-photon *in vivo* imaging of cerebrovascular dynamics during photothrombotic stroke*

**Fig. 1:**
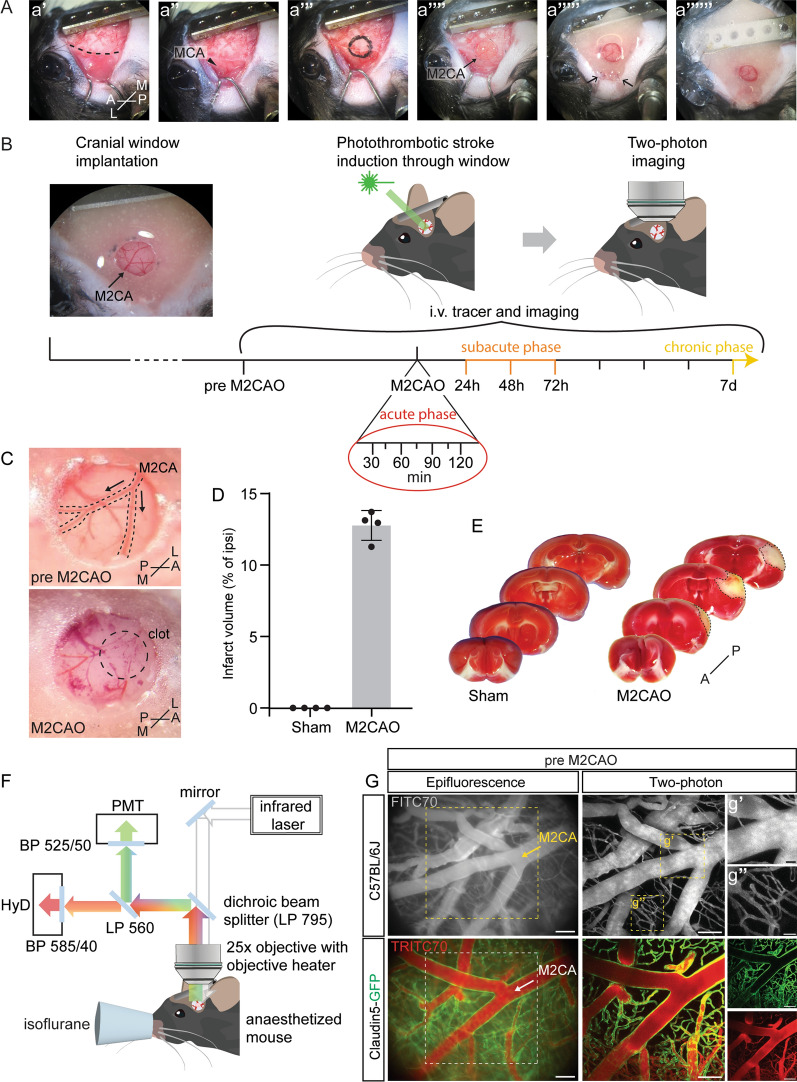
Microscope setup and imaging protocol. **A** Cranial window surgery allows optical access to the M2CA. Dashed line demarcates attachment of the temporal muscle to the cranium (a’). The arrowhead points towards the middle cerebral artery (MCA) (a’’). Position of the craniotomy is outlined (a’’’). The arrow points towards the M2CA and indicates the direction of blood flow (a’’’’). Hooks retracting the temporal muscle can be removed while the dental cement is still wet (arrows, a’’’’’). **B** Experimental outline of two-photon imaging of ischemic stroke in mice: Animals are implanted with cranial windows over the M2CA (arrow indicates direction of blood flow). Photothrombotic M2CAO is induced through the cranial window. Mice are immediately transferred to the microscope to image the acute phase of ischemic stroke. The cerebral vasculature can be followed from the acute, to the subacute and the chronic phase after ischemic stroke. Mouse schematic adapted from *scidraw.io*. **C** The M2CA before and directly after photothrombosis. Arrows indicate the direction of blood flow. **D** Quantification of TTC stainings of mouse brains 72 h after sham or stroke surgery. Sham-operated animals did not show any brain damage, while mice subjected to M2CAO had average infarct volumes of 12.7% of the ipsilateral hemisphere (n = 4 animals). **E** TTC stainings of mouse brains 72 h after sham or stroke surgery. Mice that underwent M2CAO surgery had visibly damaged tissue in the cortex and parts of the striatum (dashed line). **F** Schematic of the two-photon microscope used for dual-color imaging in the anesthetized living mouse. LP, long pass filter; BP, band pass filter; PMT, photomultiplier tube; HyD, hybrid detector. **G** Epifluorescent and two-photon images of wild-type C57BL/6 and endothelial cell reporter mice (Claudin5-GFP) intravenously injected with fluorescent tracers (FITC70, TRITC70). Scale bars (G) 100 µm; (g’-g’’) 25 µm. M-L, mediolateral axis; A-P, anterior–posterior axis

In this study, we introduce an in vivo two-photon imaging protocol that seamlessly integrates photothrombotic stroke induction via a cranial window with long-term imaging of vascular changes in the hours (acute phase) and days/weeks (subacute/chronic phase) following the ischemic event. To gain optical access to the cerebral vasculature, a chronic cranial window was implanted over the second (cortical) branch of the middle cerebral artery (M2CA) (Fig. 1A). This requires detachment and retraction of the temporal muscle from the skull. During the surgery careful attention was paid to not harm the muscle as it is involved in masticatory movement during food intake [1]. When applying the dental cement to seal the cranial window, the temporal muscle was fixed in a retracted position that did not impair its function (Fig. 1A).

After cranial window implantation and a recovery period of at least 14 days, pre M2CAO two-photon images were acquired (experimental timeline in Fig. 1B). One to three days later permanent M2CAO was induced through the cranial window by administration of rose bengal (a photoactivatable dye) and laser activation at the level of the M2CA. This will cause local endothelial cell damage, platelet aggregation and clot formation [8]. Immediately after occlusion, the animal was transferred to the microscope and the acute phase (30–120 min after occlusion) of ischemic stroke was imaged. Further, animals were imaged at 24 h, 48 h, 72 h and 7 d post ischemia in order to follow the cerebral vasculature through the subacute (24 h—72 h after occlusion) and into the beginning of the chronic phase (7 d after occlusion) (Fig. 1B).

Occlusion was induced around the first branching point of the M2CA, hence every downstream vessel segment and brain territory supplied by it were affected by a severe reduction in regional blood flow. Additional file 6: Video 1 shows the occluded M2CA (yellow dashed line) 60 min after stroke induction. Vessels in the vicinity of the M2CA show persistent blood flow (direction indicated with red arrows). Direction of blood flow in the M2CA before occlusion (arrows) and location of the clot (encircled) directly after M2CAO were recorded and is indicated in Fig. 1C. To assess lesion size 72 h after occlusion we stained brains from animals that underwent M2CAO with 2,3,5-triphenyltetrazolium chloride (TTC). TTC staining is used to discriminate between viable (red) and damaged/dead (white) tissue [3]. Mice subjected to M2CAO displayed an average infarct volume of 12.7% of the ipsilateral hemisphere (Fig. 1D). This is smaller than following laser-induced photothrombotic occlusion of the main trunk of the middle cerebral artery, where infarct volumes of 20–25% of the ipsilateral hemisphere have been reported [12, 34]. To rule out laser-mediated tissue damage, e.g., from heat generated by the laser, sham-operated animals were subjected to the same experimental procedure as M2CAO animals, except that rose bengal administration was omitted. This revealed that laser illumination by itself, without administration of rose bengal, did not induce tissue damage in sham operated mice, as analyzed by TTC staining (Fig. 1D), nor did it induce vascular damage or leakage during the first 24 h following sham surgery, as analyzed by fluorescent tracer extravasation (Additional file 3: Fig S2). Further, the infarcts in M2CAO animals were mostly confined to the cortical region (Fig. 1E, white non-viable tissue outlined with dashed lines) illustrating the selectiveness of our photothrombotic model in targeting the cortical branch of the M2CA. Taken together, any damage observed following the M2CAO surgery can be attributed to the ischemic insult.

The microscope setup used in this protocol enables dual-color imaging with single-wavelength two-photon excitation (Fig. 1F). The excitation light in the infrared light spectrum is guided to the anesthetized animal via mirrors and a dichroic beam splitter which subsequently reflects the emitted light in the visible light spectrum. Using a combination of long-pass and band-pass filters, fluorescent signals in the green and red light spectrum are separated before detection with hybrid detectors and photomultiplier tubes, respectively. Epifluorescent images were used for orientation and navigation to the same imaging position during subsequent imaging sessions (Fig. 1G). Vessel perfusion and BBB breakdown were visualized using i.v. administered green- and red-shifted fluorescent dextrans (FITC70 and TRITC70, respectively) in wild-type C57BL/6 and endothelial reporter mice (expressing GFP under the Claudin5 promoter).

### Quantification of vascular leakage and perfusion in the acute phase after vessel occlusion

**Fig. 2:**
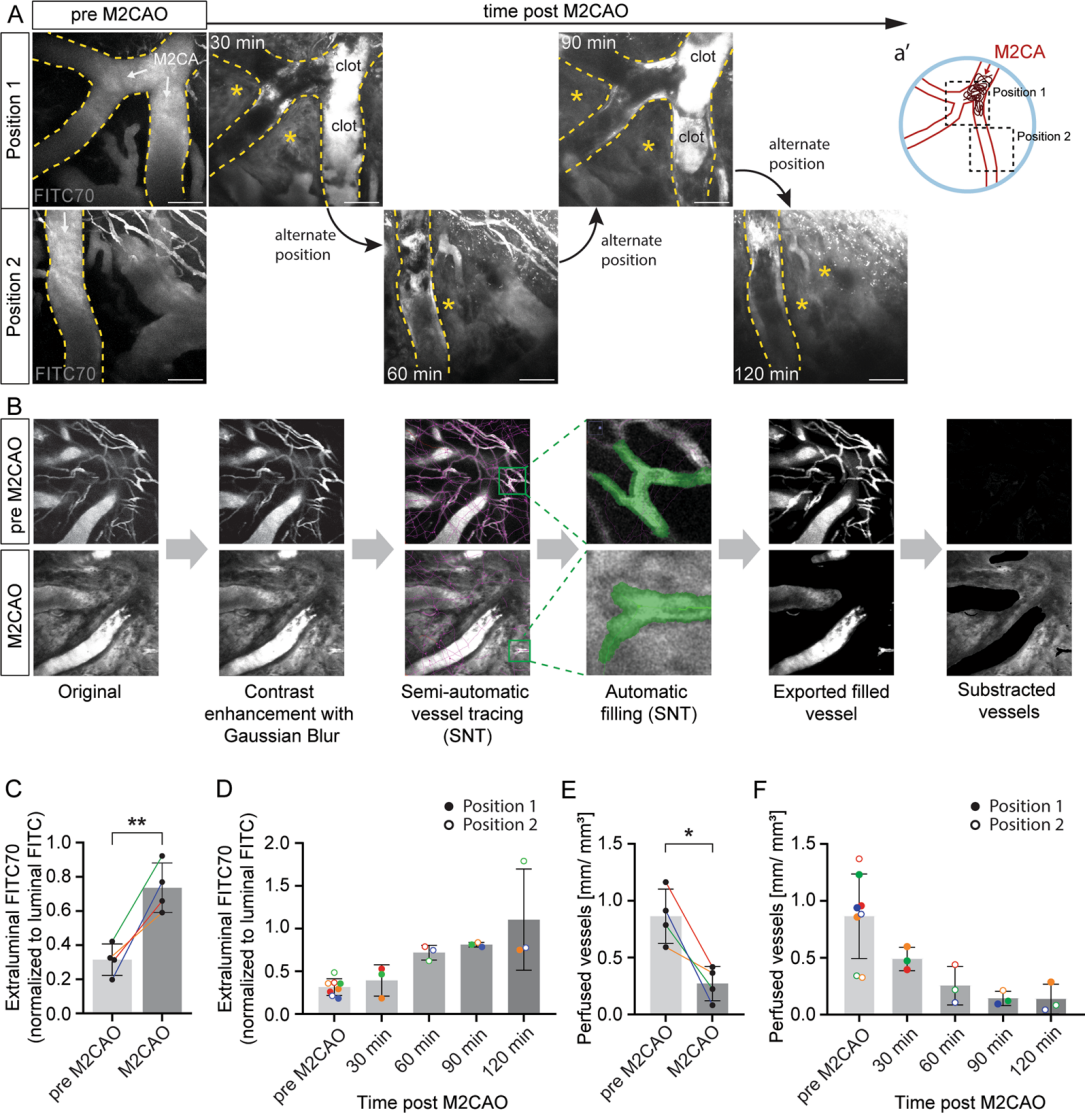
Vascular leakage and perfusion in the acute phase after ischemic stroke. **A** Maximum intensity projections of two-photon imaged z-stacks of FITC70 at two alternating positions (schematic in a’) before and at different time points after M2CAO. Landmark vessels for orientation are outlined with dashed lines. M2CA and direction of blood flow are indicated (arrows). The location of the clot is marked. Asterisks indicate extravasated, parenchymal FITC70. Scale bars 100 µm. **B** Flow chart of three-dimensional (3D) leakage analysis using Simple Neurite Tracer (SNT) in ImageJ/Fiji. **C** Average FITC70 leakage before and after M2CAO. n = 4 animals. Two positions were imaged per animal. Colored lines indicate individual animals. **p < 0.01 (two-tailed, paired *t*-test). **D** FITC70 extravasation at different time points post M2CAO. n = 4 mice. Colored symbols indicate individual animals. **E** Average perfused vessel density before and after M2CAO. n = 4 animals. Two positions were imaged per animal. Colored lines indicate individual animals. *p < 0.05 (two-tailed, paired *t*-test). **F** Perfused vessel density at different time points post M2CAO. n = 4 mice. Colored symbols indicate individual animals

Wild-type C57BL/6 mice were subjected to M2CAO and imaged before (pre M2CAO) and at different timepoints during the first 120 min after M2CAO (Fig. 2A). Images were acquired at two different positions: Position 1, at the level of the M2CA bifurcation where the clot was induced and Position 2, at a proximal segment of the M2CA downstream of the occlusion (schematic illustration shown in Fig. 2a’). The imaging pipeline includes alternating between the two positions as outlined in Fig. 2A. Diminished blood flow is visible as a shadow within the M2CA (Fig. 2A, post M2CAO). Additional file 7: Video 2 shows a timelapse of the occluded M2CA 75 min after induction of M2CAO. In this video, the red arrowhead marks a fully occluded vessel segment, while the red arrow points at a vessel segment that remained partially open at this time point and displayed diminished blood flow. During pre M2CAO imaging, the FITC70 signal was confined to the vessel lumen (illustrated with dashed lines), while already at 30 min post M2CAO, extraluminal FITC70 signal was observed in the brain parenchyma (Fig. 2A, asterisks) as well as in the subarachnoid space (Additional file 2: Fig S1A) due to vascular leakage. Segmentation of the extra- and intraluminal FITC70 signal was achieved using the Simple Neurite Tracer plugin (SNT, [22]) in ImageJ/Fiji to semi-automatically trace the vessels within the acquired z-stack and to fill the vessel lumen (Fig. 2B, for more details see Methods). The filled vessels, i.e., the intraluminal FITC70 signal, were exported and subtracted from the original file resulting in the separation of the extraluminal FITC70 signal (Fig. 2B). Note that the high extraluminal signal after M2CAO makes edge-detection and thus automatic filling of the vessel lumen difficult for the SNT algorithm, hence, vessel filling after M2CAO was performed under human supervision and corrected when necessary. Extraluminal FITC70 was quantified throughout the acquired z-stack by normalizing the extraluminal fluorescence signal to the intraluminal signal. These analyses revealed significantly increased average FITC70 extravasation during the first 120 min post M2CAO compared to pre M2CAO (Fig. 2C, p = 0.0093; two-tailed, paired *t*-test; n = 4 animals). Analysis of extraluminal FITC70 signal in individual animals over time, showed that vascular leakage occurred early and gradually increased with time during the first hours after induction of M2CAO (Fig. 2D). Due to the curvature of the brain, image planes close to the meninges also partly detected FITC70 signal in the subarachnoid space which consequently was included in the vascular leakage analysis above. However, we demonstrate that segmentation of the brain parenchymal and subarachnoid signal is possible (Additional file 2: Fig S1A, B). This illustrates that vascular leakage after ischemic stroke can be visualized and quantified three-dimensionally with two-photon imaging.

Quantification of perfused vessel density by tracing of intraluminal FITC70 signal throughout the acquired z-stacks revealed a significant reduction in the average density of perfused vessels in the ischemic region during the first 120 min post M2CAO compared to pre M2CAO (Fig. 2E, p = 0.0216; two-tailed, paired *t*-test; n = 4 animals). This is contrary to what we found when analyzing vascular leakage, where extraluminal tracer increased over time (Fig. 2C). This negative correlation between leakage and perfused vessel density is even more apparent when analyzing each individual animal (Fig. 2D, F). Taken together, we have established a protocol to follow vascular leakage and tissue perfusion during stroke progression in the same animal in real-time.

### Dissecting BBB breach along the arteriovenous axis

**Fig. 3:**
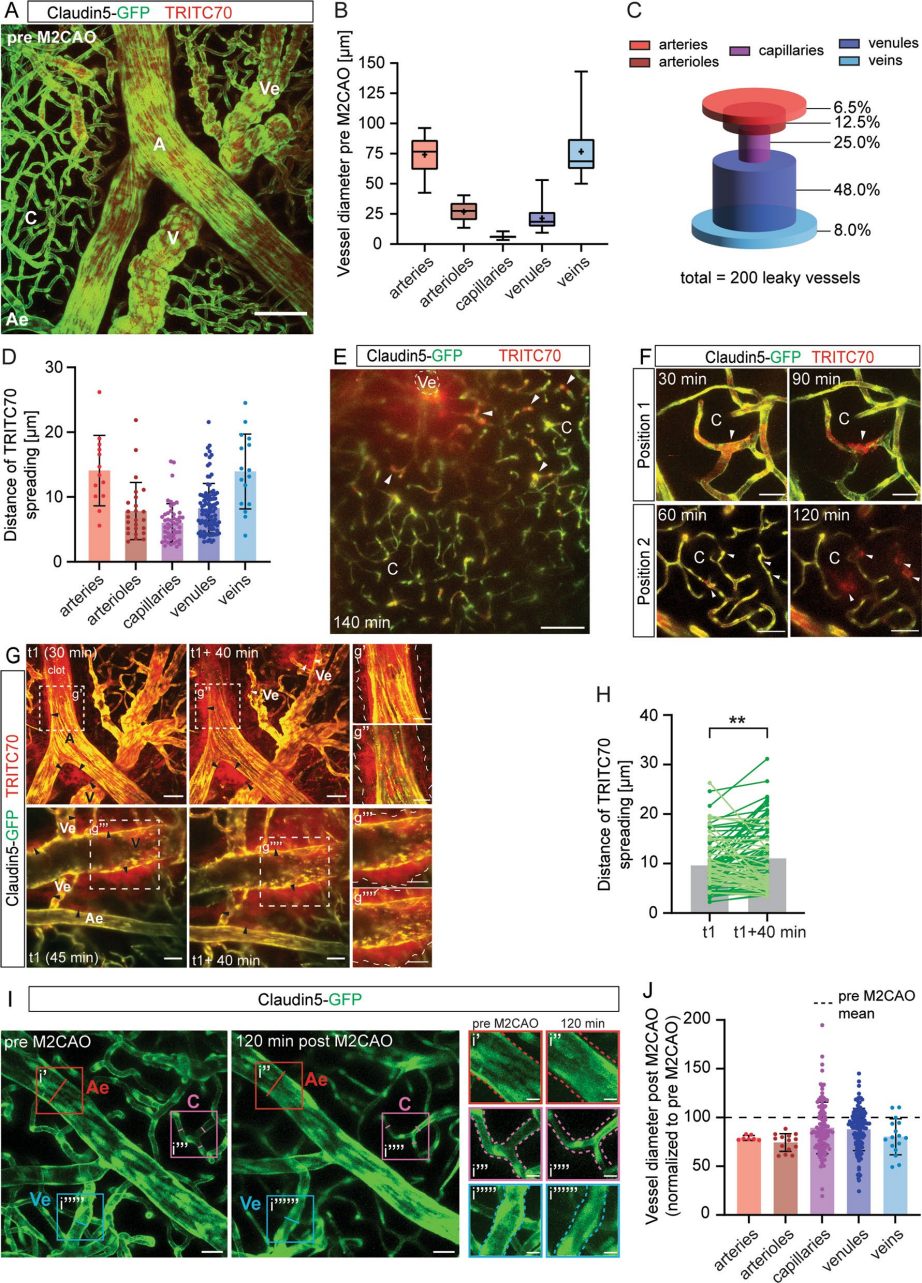
Vessels along the arteriovenous (AV) axis during the acute phase of ischemic stroke. **A** Maximum intensity projection of cerebral vessels in Claudin5-GFP reporter mice depicting endothelial cells (green) and TRITC70 signal (red). A, artery; Ae, arteriole; C, capillaries; Ve, venule; V, vein. Scale bar 100 µm. **B** Pre M2CAO vessel diameter along the AV axis. **C** Distribution of leaky vessels along the AV axis during the acute phase after occlusion. n = 200 leaky vessels from 4 animals. **D** Quantification of TRITC70 extravasation from leaky vessels during the acute phase after occlusion. n = 200 leaky vessels from 4 animals. **E** Maximum intensity projection of the capillary (C) bed 140 min post M2CAO. TRITC70 leakage around an ascending venule (Ve, lumen outlined) and capillaries (arrowheads) can be observed. Scale bar 100 µm. **F** Example images of leaky capillaries at 90 min and 120 min post M2CAO (arrow heads). Scale bars 25 µm (Position 1) and 50 µm (Position 2). **G** Images as in (A) during the acute phase after M2CAO. Sites of leakage are marked with arrowheads. TRITC70 leakage distance is demarcated with dashed lines (g’-g’’’’). t1, time point 1; scale bars (G) 50 µm; scale bars (g’-g’’’’) 25 µm. **H** Quantification of TRITC70 extravasation from leaky vessels at t1 and t1 + 40 min; increased (dark green) and decreased (light green) leakage from individual vessels indicated, n = 100 leaking vessels from 4 animals, **p < 0.005 (two-tailed, paired t-test). **I** Example image of vasoconstriction in the peri-infarct region 120 min after M2CAO. Arterioles (Ae) marked in red; capillaries (C) marked in purple; venules (Ve) marked in blue. Scale bars (I) 25 µm, (i’-i’’’’’’) 10 µm. **J** Relative vessel diameter changes after M2CAO. n (artery) = 7; n (arteriole) = 14; n (capillary) = 113; n (venule) = 128; n (vein) = 16; from 4 animals

The physiological function of the BBB lies in selectively restricting blood-born molecules and cells from entering the brain parenchyma. At the capillary level, where nutrient and oxygen exchange between the brain and the blood occurs, properties of the BBB have been extensively studied (for review see e.g. [7, 35]. Less is known about the barrier in other segments of the vascular tree; indeed, it has only recently been suggested that the manifestation of the barrier might reside within different cell types in different vascular segments [2]. As our analyses demonstrated no leakage of fluorescent tracer from any cerebral vessel in naïve animals (Figs. 1G, 2A (pre M2CAO), 3A, Additional file 3: Fig S2), we here define BBB broadly to include all cerebrovascular vessel classes from capillaries to arteries and veins. To establish a protocol to define stroke-induced BBB breach along the AV axis, we made use of Claudin5-GFP mice, which selectively express cytosolic GFP in endothelial cells. This allowed us to distinguish between arteries (A), arterioles (Ae), capillaries (C), venules (Ve), and veins (V) on account of their endothelial cell morphology (Fig. 3A and their diameter, see Methods for details). Quantifying the diameter of the different vessel classes revealed average vessel diameters of arteries, 74.1 ± 14.5 µm (n = 29); arterioles, 26.8 ± 8.0 µm (n = 28); capillaries, 6.1 ± 1.2 µm (n = 236); venules, 21.3 ± 8.9 µm (n = 190); and veins, 76.7 ± 25.0 µm (n = 29)(Fig. 3B).

Using the two-photon setup we developed a protocol to analyze BBB breakdown along the AV axis where parenchymal tracer accumulation was attributed to the nearest vessel segment and quantified (Additional file 4: Fig S3). Defining the identity of the permeable vessels during the first 120 min after M2CAO (n = 200 vessels from 4 animals) revealed that about half of the analyzed leaky vessels were venules (48%), a quarter were capillaries (25%), 12.5% were arterioles, while arteries and veins accounted for only 6.5% and 8%, respectively (Fig. 3C). It should be noted, that even though the proportion of leaky arteries and veins were low in our analysis, their contribution to leakage might still be substantial. To determine the contribution of various vessels to leakage, we measured the distance of TRITC70 signal from the vessel wall into the parenchyma as a proxy for the amount of TRITC70 that had leaked into the extravascular space from that vessel (Fig. 3D). We found that TRITC70 signal was detected on average 14.1 ± 5.4 µm and 13.9 ± 5.8 µm from the vessel wall of arteries and veins, respectively. Figure 3E shows leakage of TRITC70 from an ascending venule and from capillaries. The distance of TRITC70 spreading from the vessel wall of capillaries was shorter compared to the other vessel classes (6.0 ± 3.0 µm µm; Fig. 3D, F); yet this is a substantial distance considering the capillary diameter and thus contained blood volume. As brain tissue is nearly exclusively perfused by capillaries, they may be attributed major sites of leakage after M2CAO. Leakage within the capillary bed in the first two hours after vessel occlusion can be seen in Additional file 8: Video 3.

We showed that using this protocol it is possible to assess progression of leakage from single vessels in real-time by comparing parenchymal TRITC70 spreading from the vessel wall at t1 with that at t1 + 40 min (Fig. 3G). An increase in the distance of TRITC70 signal from the vessel wall into the parenchyma was interpreted as persistent leakage, whereas a decrease indicated a reduction or even stopped leakage. We found that overall, the TRITC70 signal increased between t1 and t1 + 40 min (*p* = 0.0047; two-tailed, paired *t*-test; n = 100 vessels from 4 animals, Fig. 3H) and that 67% of the vessels analyzed continued to leak during this time frame (dark green lines, Fig. 3H).

Also, we demonstrated that this setup is a powerful tool to assess the effect of ischemia on vasoconstriction/dilation along the AV axis. Determining the vessel diameter pre M2CAO (Fig. 3B, I) enabled us to observe changes occurring with time after M2CAO (Fig. 3I). We found a global constriction of the vascular tree which was most pronounced in the arterial system (26.5% and 21.8% average vessel diameter decrease in arterioles and arteries, respectively). A similar, but less pronounced effect, was observed in the venous system (20.6% and 13.1% average vessel diameter decrease in veins and venules, respectively) and in capillaries (11.3% reduction) (Fig. 3J, Additional file 5: Fig S4).

### Longitudinal studies on vascular remodeling after ischemia

**Fig. 4:**
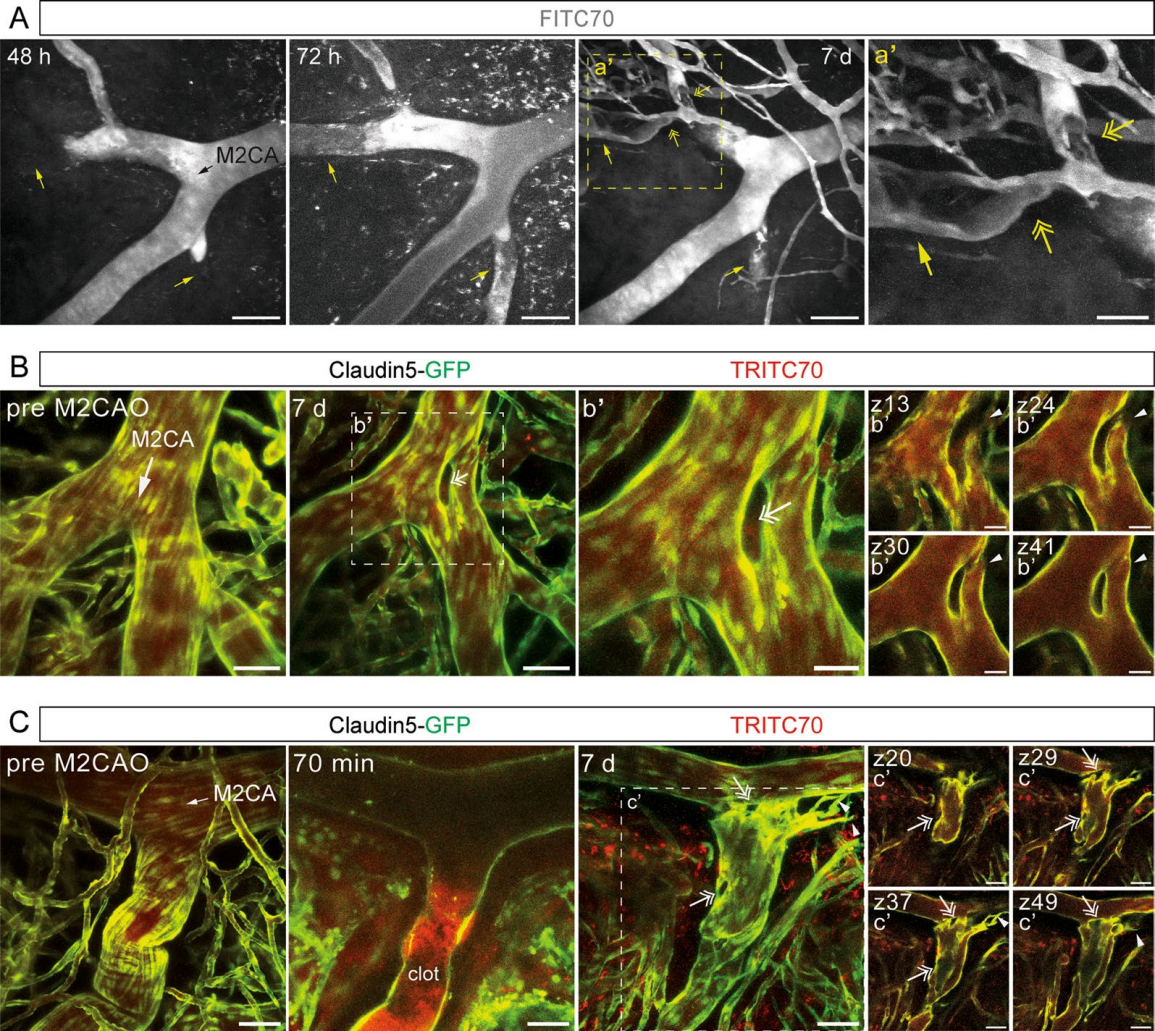
Longitudinal imaging of the cerebral vasculature during the subacute and chronic phase after ischemic stroke. **A** Maximum intensity projections of two-photon imaged z-stacks of FITC70 signal in M2CA field of view 48 h, 72 h and 7 d post M2CAO, respectively. Black arrow indicates direction of blood flow; yellow arrows indicate vessels with blocked (48 h) and re-established blood flow at transition to chronic phase (72 h/7 d); yellow two-headed arrows indicate putative residual clots; scale bars 100 µm. **B**, **C** Maximum intensity projection of M2CA field of view in Claudin5-GFP reporter mice depicting endothelial cells (green) and TRITC70 signal (red) before and after M2CAO; (b', c') depict individual z-planes. White arrows indicate direction of blood flow; arrowheads indicate loop formed in bifurcation (b') and angiogenic sprouting (c'); two-headed arrows indicate intussusceptive pillars; scale bars (B, C, c’) 50 µm; scale bars (b’) 25 µm

The first two weeks after an ischemic infarct have been shown to be a time of increased vascular plasticity in which blood vessels in the tissue undergo major changes, thus affecting tissue perfusion and blood flow [41]. However, current knowledge on the plastic changes occurring at the level of single vessels during this time frame remains limited. Application of the two-photon imaging protocol permitted data collection on remodeling and blood flow dynamics of single vessel segments 48 h to 7 d following M2CAO (Fig. 4). Using FITC70 we were able to visualize blood flow at single vessel resolution during the transition from the subacute to the chronic phase after onset of ischemia. We identified blood vessels where blood flow was blocked 48 h post M2CAO but re-established one day later at 72 h post M2CAO (arrows, Fig. 4A). At the beginning of the chronic phase at 7 d post M2CAO, blood flow in the previously occluded vessel had been profoundly modulated (two-headed arrows). It appeared as if the blood was bypassing an obstacle, potentially the residual clot, which led to a stark narrowing of the vessel lumen (Fig. 4A). This is likely to affect downstream vessels and the brain areas supplied by it. Identifying vulnerability of certain blood vessels to prolonged obstruction and factors affecting it is of high clinical relevance.

Further, making use of Claudin5-GFP reporter mice [38] in combination with TRITC70 for visualization of blood flow allowed us to combine information about the perfusion status of a vessel and the endothelial cell organization in the vessel wall. Figure 4B shows the M2CA of a Claudin5-GFP mouse before M2CAO as well as 7 d after M2CAO. We found that a week after the ischemic infarct an intussusceptive pillar had formed at the branching point of the M2CA, thus forming a loop between the trunk of the M2CA and the vessel segment that branched off it. An intussusceptive pillar in this location can be expected to perturb laminar blood flow through the M2CA and cause an increase in wall shear stress which could have long-term consequences for the vessel. Figure 4C depicts the M2CA of a different individual in which the branch of the M2CA, which was heavily occluded after the infarct, underwent major reconstruction 7 d post M2CAO. We found multiple intussusceptive pillars (two-headed arrow), vascular sprouts (arrowheads) and an intertwining network of vessels where a single large branch used to be. Two-photon imaging after M2CAO can thus be used to follow reperfusion, revascularization, and remodeling of the cerebral vasculature days to weeks after the ischemic insult in order to gain insights into the dynamic processes underlying adaptation to stroke pathology.

## Discussion

In this study we have established an in vivo two-photon imaging and analysis pipeline which enables studies on cerebrovascular dynamics in real-time at single vessel resolution following large artery ischemic stroke in mice. For this we adapted the well-established protocol of photothrombotic middle cerebral artery occlusion (MCAO) [33, 34, 36] and induced thrombosis in the cortical branch of the MCA (M2CAO) through a chronically implanted cortical window. In photothrombotic models of ischemic stroke, light activation of a photoactivatable dye causes generation of short-lived reactive oxygen species, leading to local endothelial cell damage and clot formation specifically in the superficial artery where the laser is directed. Disruption of the BBB occurs locally and in the downstream vascular bed as a direct consequence of the ischemic insult [24, 39], making it well-suited for BBB research. BBB disruption is a hallmark of ischemic stroke in humans and is seen in many experimental models of ischemic stroke (including in embolic and transient models), although the extent, time course and potentially the mechanism of leakage might differ between the models due to the different ways of occluding the supplying artery. We found that the M2CAO infarcts were mainly restricted to the cortical region and that they were smaller than infarcts induced by photothrombotic occlusion of the first bifurcation of the MCA [34]. This was to be expected considering the smaller downstream brain region supplied by the cortical MCA branch and illustrates the selectivity of the model.

To be able to quantify vascular leakage, we employed a semi-automated segmentation algorithm [22] that separated the fluorescent tracer signal in the vessel lumen from the extraluminal signal. Using this method, we demonstrate that temporal dynamics of vascular leakage can be followed in the 3D space from superficial large vessels transversing the subarachnoid space to deeper residing small vessels. This extends previous intravital research which has been restricted to observations in single imaging planes or small, predefined regions of interest close to the vasculature [4, 14]. We show that our analysis protocol allows for detailed examination of dynamic vascular leakage in real-time, commencing as early as 30 min after ischemic stroke onset. This is of particular importance, because traditional ex vivo analyses do not support dynamic studies of vascular integrity nor studies on very acute vascular leakage due to restrictions with the required circulation time of the chromatic compound, yet the acute phase of ischemic stroke is a highly critical period in ischemic stroke patients, where the ‘onset-to-treatment’ time is one of the strongest predictors of clinical outcome [31].

In addition, we demonstrated that dual-color imaging of fluorescently labeled blood flow in endothelial cell reporter mice enables examination of BBB breach and hemodynamic parameters along the AV axis. During the acute phase of ischemic stroke, we conducted an analysis to quantify the extent of vascular leakage across various vessel types. Our findings show that, in absolute terms, the amount of leakage is higher the larger the vessels. Yet, we found that in proportion to vessel diameter and blood volume, leakage from capillaries was in fact the largest. This is in line with the findings presented in an extensive ex vivo study, in which BBB breach was measured and ascribed to vessel types at 4 h and 24 h in murine brains subjected to three different experimental ischemic stroke models [18]. This emphasizes the validity and usefulness of our intravital two-photon imaging and analysis protocol, as it can detect hemodynamic changes in single vessels in real-time. It is conceivable that different vessel types, in different locations might be more susceptible to BBB breakdown during certain phases of stroke pathology, thus the protocol presented here could assist future research aiming to uncover the temporal dynamics of BBB leakage along the AV axis.

Further insights into the cellular and molecular mechanisms underlying BBB breakdown, vascular regrowth or scar formation after an ischemic event, can be made utilizing the vast number of genetically modified mouse lines available. This includes mice with fluorescently labeled astrocytes, which have recently been employed to study the involvement of reactive astrocytes in revascularization and vascular plasticity after ischemic stroke by intravital two-photon imaging [20, 42]. Moreover, conditional knockout or overexpressing mouse lines can be deployed to dissect the role certain proteins or signaling pathways play in BBB regulation during stroke progression. Importantly, this imaging protocol offers the possibility to study the effect of drugs and drug candidates on BBB breakdown at high spatial and temporal resolution. This includes studies with recombinant tissue plasminogen activator (r-tPA), the only approved thrombolytic drug for treatment of ischemic stroke. Unfortunately, a common side effect of r-tPA treatment is hemorrhagic transformation which severely aggravates stroke pathology [32, 44]. Intravital imaging of the cerebrovasculature after r-tPA administration will contribute valuable information to why treatment with r-tPA can lead to such serious side effects.

Lastly, using this method we were able to longitudinally study the same blood vessels across days during the transition from the acute, to the subacute and chronic phase after ischemic onset. We demonstrate that we can detect different forms of (maladaptive) vascular remodeling in the previously occluded blood vessels in the chronic phase, including intussusceptions, angiogenic sprouting and entangled vessel networks. Extending this line of research has potential clinical relevance, as it is still a topic of open debate what causes the high number of patients suffering from recurrent strokes [11]. One hypothesis put forth is that, in addition to stroke-associated risk factors, maladaptive vascular remodeling increases the vulnerability of blood vessels to recurrent ischemic events. Hence, understanding these processes by detailed intravital investigation might lead to the discovery of new targets for therapeutic intervention.

## Conclusion

In this study we show that in vivo two-photon imaging is a powerful tool to investigate BBB breakdown and vascular remodeling after photothrombotic ischemic stroke. Intravital imaging can be applied to delineate the dynamics of vascular leakage in the critical early hours after vessel occlusion as well as to longitudinally follow the response of the cerebral vasculature to ischemia during the subacute and chronic phase after stroke onset. The analysis protocol that we present here enables analysis of BBB breach in the 3D space along the AV axis, thereby allowing dissection of the contribution of single vessels to stroke progression. The relevance and importance of the presented imaging and analysis protocol lies within its potential to elevate BBB research from static, single timepoint analyses in large cohorts of animals to dynamic, longitudinal analyses in individual animals which allows correlation between different parameters during stroke progression and functional outcome.

### Supplementary Information

**Additional file 1: Table S1.** List of mice used in this study.

**Additional file 2: Figure S1.** Segmentation of intracranial vascular leakage after M2CAO. (A) Pseudocolored single imaging plane close to the pial surface showing the crude segmentation (yellow dashed line) of the brain parenchyma (green) and the subarachnoid space (blue) based on the presence of pial capillaries (orange arrow) before and 30 min after M2CAO. Scale bar 100 µm (B) Segmentation of the subarachnoid space and the brain parenchyma through the z-stack and normalization of the extravascular signal to the intraluminal signal within the respective region-of-interest showed a similar leakage pattern in the brain parenchyma and the subarachnoid space over time.

**Additional file 3: Figure S2.** Examination of cerebrovascular leakage after sham surgery. Similar to M2CAO induction, the M2CA was illuminated with a laser for 20 min during the sham surgery but PBS was injected instead of rose bengal which does not result in intravascular clot formation. The cerebral vessels were imaged 60 min, 90 min and 24 h post sham surgery. During those timepoints, we did not find extravasation of the fluorescent tracer TRITC70 in arteries (A), veins (V) or capillaries (C). Additionally, we could verify that the endothelial GFP signal was not affected by the laser illumination during the stroke/sham surgery. Scale bars 50 µm.

**Additional file 4: Figure S3.** Leaky artery, arteriole, capillary, venule and vein during the acute phase of ischemic stroke. Maximum intensity projection of cerebral vessels in Claudin5-GFP reporter mice depicting endothelial cells (green, middle row) and TRITC70 signal (red, bottom row) 120 min after M2CAO; merged image, top row. TRITC70 extravasation from the vessel lumen into the parenchyma is outlined with dashed lines. TRITC70 is not visible in the green channel, yet the Claudin5-GFP signal can leak into the red channel when GFP expression is very high (asterisks). Scale bars (artery, arteriole, venule, vein) 25 µm, scale bars (capillary) 10 µm.

**Additional file 5: Figure S4.** Vessel constriction within the capillary bed. Maximum intensity and side projections of the capillary bed before and 140 min after M2CAO. Global constriction of the capillaries can be observed (indicated with arrowheads at clearly identifiable vessel segments). Scale bars 100 µm.

**Additional file 6: Video 1.** M2CA 60-65 min after ischemic stroke (imaged with epifluorescent camera). Stalled blood flow due to vessel occlusion can be seen in the M2CA (demarcated with yellow dashed line). Persistent blood flow can be seen in non-occluded vessels in close proximity to the M2CA. Direction of blood flow is indicted with arrows. Claudin5-GFP in grey. Scale bar 100 µm.

**Additional file 7: Video 2.** M2CA 75-82 min after ischemic stroke. The arrowhead points towards a fully occluded branch of the M2CA, while the arrow points towards a partially occluded branch. The vessel is outlined with dashed lines. The grey arrows indicate the direction of blood flow. Note the high amount of extravasated FITC70 in the parenchyma. Scale bar 50 µm.

**Additional file 8: Video 3.** Capillary bed 33-130 min after ischemic stroke (Scale bar 50 µm). The arrowheads point towards (temporarily) leaking capillaries. The capillary marked with the magenta arrowhead is magnified to the right (scale bars 10 µm). Claudin5-GFP in green, TRITC70 in red. Note the increase of diffuse parenchymal TRITC70 over time.

## Data Availability

The datasets used and/or analyzed during the current study are available from the corresponding author on reasonable request.

## Abbreviations

3D: Three-dimensional
A: Artery
Ae: Arteriole
A/P: Anterior–posterior
AV: Arteriovenous
BP: Band-pass
C: Capillary
FITC70: 70 kDa fluorescein isothiocyanate
GFP: Green fluorescent protein
HyD: Hybrid detector
i.v.: Intravenous
LP: Long-pass
M2CA: Second segment of the middle cerebral artery
M2CAO: Occlusion of the second segment of the middle cerebral artery
MCA: Middle cerebral artery
MCAO: Middle cerebral artery occlusion
M/L: Medio-lateral
MRI: Magnetic resonance imaging
NA: Numerical aperture
PBS: Phosphate buffered saline
PMT: Photomultiplier tube
r.o.: Retroorbital
s.c.: Subcutaneous
TRITC70: 70 kDa tetramethylrhodamine
TTC: 2,3,5-Triphenyltetrazolium chloride
V: Vein
Ve: Venule
