# Supplementary material for: Analysis of ischemic stroke-mediated effects on blood–brain barrier properties along the arteriovenous axis assessed by intravital two-photon imaging
Source: Fluids Barriers CNS. 2024 Apr 15;21:35. doi: 10.1186/s12987-024-00537-5 (PMC11017501; doi:10.1186/s12987-024-00537-5)
Supplement: Supplementary file 1 — Additional file 1: Table S1. List of mice used in this study. [file 12987_2024_537_MOESM1_ESM.docx]

**Additional file 1: table S1.** List of mice used in this study.

| **Mouse** | **Sex** | **Genotype** | **Age at M2CAO/sham induction** | **Experiment used in** |
| --- | --- | --- | --- | --- |
| **#1** | male | C57BL/6 | 3 m(onths)  22 d(ays) | Infarct volume (Fig. 1D, M2CAO group) |
| **#2** | female | C57BL/6 | 3 m 22 d | Infarct volume (Fig. 1D, M2CAO group) |
| **#3** | female | C57BL/6 | 3 m 22 d | Infarct volume (Fig. 1D, M2CAO group) |
| **#4** | female | C57BL/6 | 3 m 22 d | Infarct volume (Fig. 1D, M2CAO group) |
| **#5** | female | C57BL/6 | 2 m 10 d | Infarct volume (Fig. 1D,  sham group) |
| **#6** | female | C57BL/6 | 2 m 10 d | Infarct volume (Fig. 1D,  sham group) |
| **#7** | female | C57BL/6 | 2 m 10 d | Infarct volume (Fig. 1D,  sham group) |
| **#8** | female | C57BL/6 | 2 m 10 d | Infarct volume (Fig. 1D,  sham group) |
| **#9** | male | C57BL/6 | 3 m 24 d | Leakage, perfusion (Fig. 2C-F; Suppl. Video 2)  Vascular remodeling (Fig. 4A) |
| **#10** | female | C57BL/6 | 5 m 0 d | Leakage, perfusion (Fig. 2C-F) |
| **#11** | male | C57BL/6 | 3 m 5 d | Leakage, perfusion (Fig. 2C-F) |
| **#12** | female | C57BL/6 | 4 m 5 d | Leakage, perfusion (Fig. 2C-F) |
| **#13** | female | Claudin5-GFP | 3 m 24 d | Leakage AV axis (Fig. 3B-D, H, J), vascular remodeling (Fig. 4B) |
| **#14** | male | Claudin5-GFP | 5 m 20 d | Leakage AV axis (Fig. 3B-D, H, J) |
| **#15** | female | Claudin5-GFP | 6 m 17 d | Leakage AV axis (Fig. 3B-D, H, J), vascular remodeling (Fig. 4C) |
| **#16** | male | Claudin5-GFP | 3 m 18 d | Leakage AV axis (Fig. 3B-D, H, J) |
| **#17** | female | Claudin5-GFP | 6 m 22 d | Leakage after sham (Suppl. Fig. 2) |
| **#18** | female | Claudin5-GFP | 9 m 17 d | Blood flow after M2CAO in M2CA vicinity (Suppl. Video 1) |
| **#19** | male | Claudin5-GFP | 5 m 19 d | Leakage in capillaries (Suppl. Video 3) |
