# Supplementary figures and images for: Analysis of ischemic stroke-mediated effects on blood–brain barrier properties along the arteriovenous axis assessed by intravital two-photon imaging
Source: Fluids Barriers CNS. 2024 Apr 15;21:35. doi: 10.1186/s12987-024-00537-5 (PMC11017501; doi:10.1186/s12987-024-00537-5)

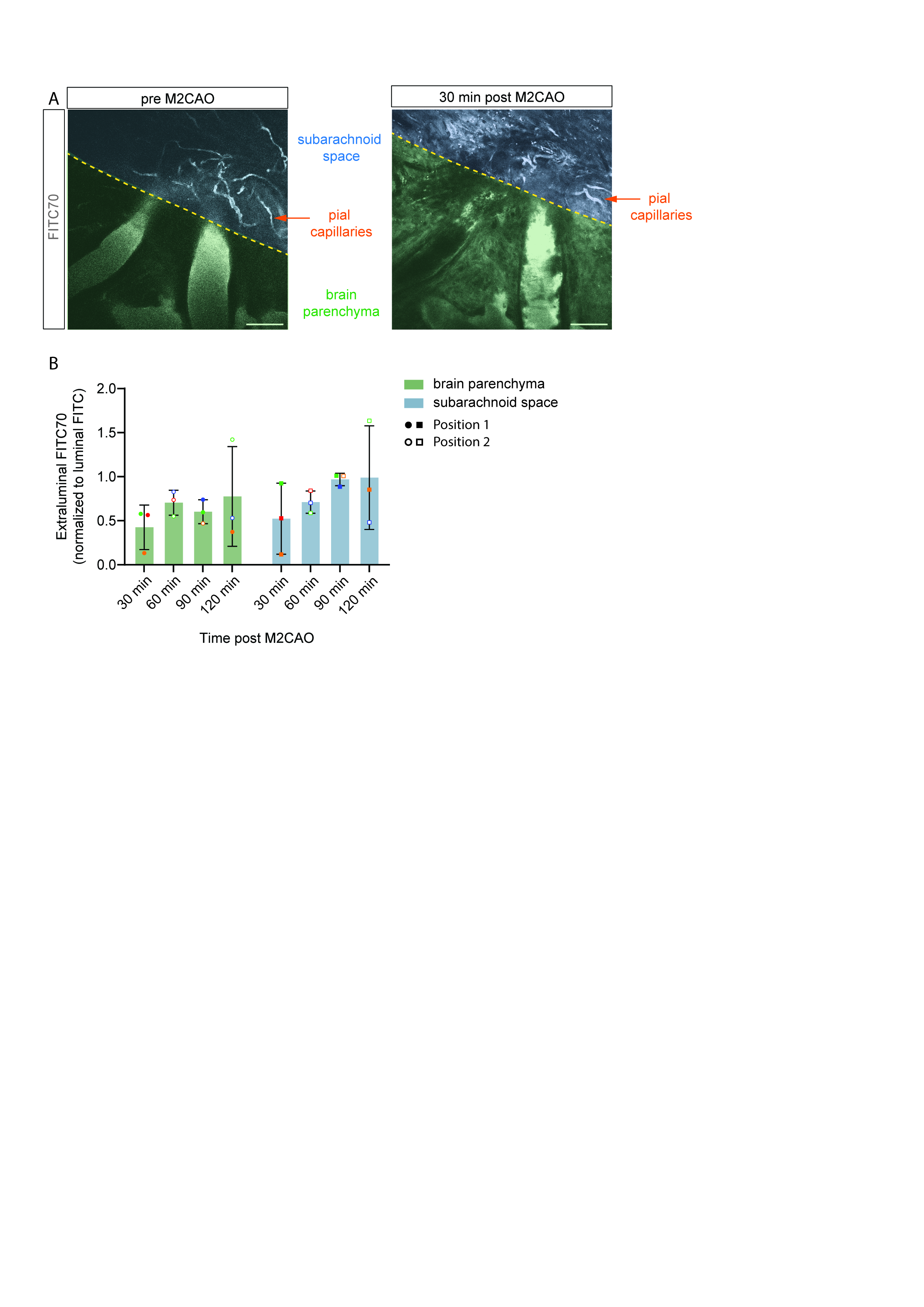

Supplement: Supplementary file 2 — Additional file 2: Figure S1. Segmentation of intracranial vascular leakage after M2CAO. (A) Pseudocolored single imaging plane close to the pial surface showing the crude segmentation (yellow dashed line) of the brain parenchyma (green) and the subarachnoid space (blue) based on the presence of pial capillaries (orange arrow) before and 30 min after M2CAO. Scale bar 100 µm (B) Segmentation of the subarachnoid space and the brain parenchyma through the z-stack and normalization of the extravascular signal to the intraluminal signal within the respective region-of-interest showed a similar leakage pattern in the brain parenchyma and the subarachnoid space over time. [file 12987_2024_537_MOESM2_ESM.tif]

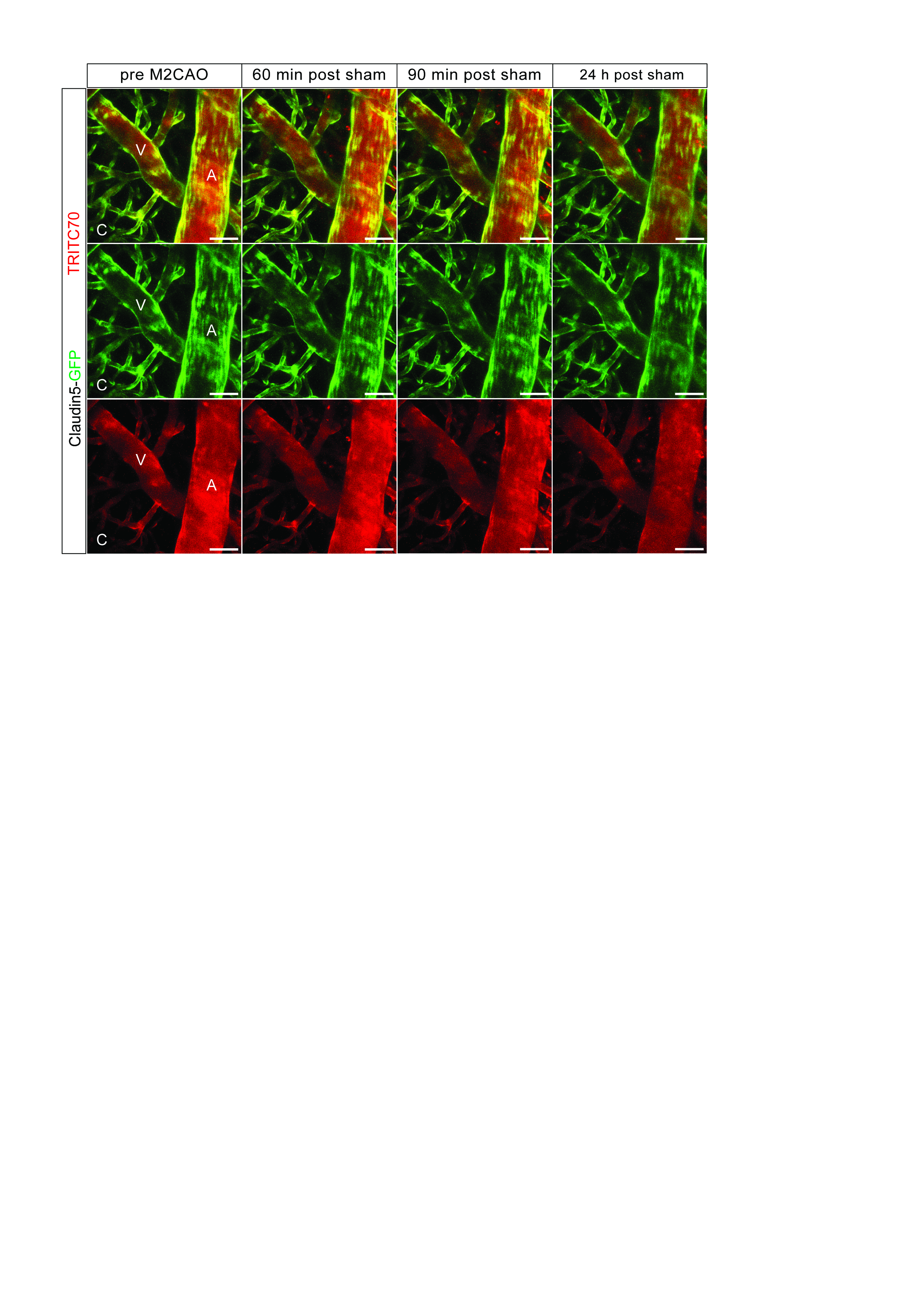

Supplement: Supplementary file 3 — Additional file 3: Figure S2. Examination of cerebrovascular leakage after sham surgery. Similar to M2CAO induction, the M2CA was illuminated with a laser for 20 min during the sham surgery but PBS was injected instead of rose bengal which does not result in intravascular clot formation. The cerebral vessels were imaged 60 min, 90 min and 24 h post sham surgery. During those timepoints, we did not find extravasation of the fluorescent tracer TRITC70 in arteries (A), veins (V) or capillaries (C). Additionally, we could verify that the endothelial GFP signal was not affected by the laser illumination during the stroke/sham surgery. Scale bars 50 µm. [file 12987_2024_537_MOESM3_ESM.tif]

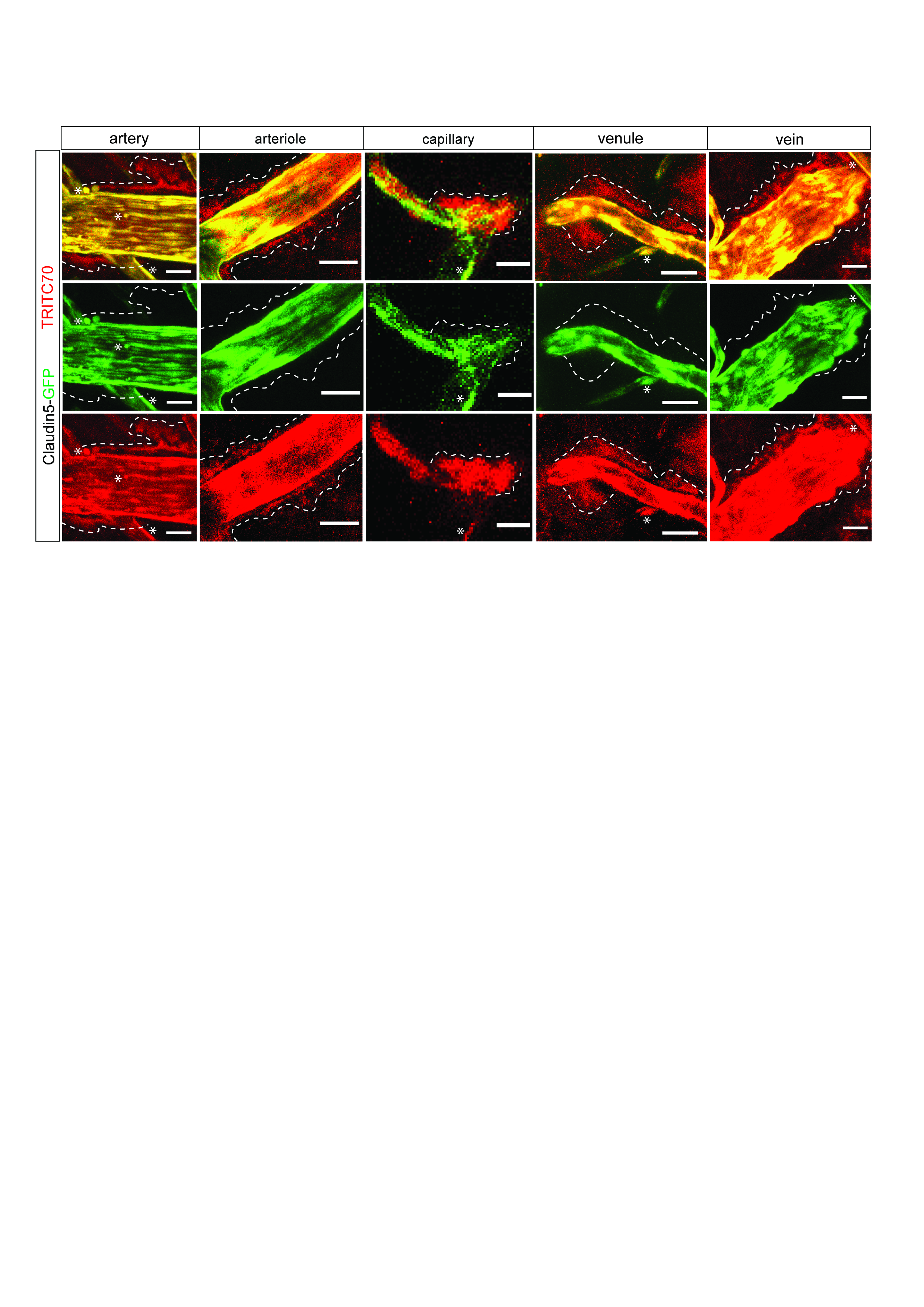

Supplement: Supplementary file 4 — Additional file 4: Figure S3. Leaky artery, arteriole, capillary, venule and vein during the acute phase of ischemic stroke. Maximum intensity projection of cerebral vessels in Claudin5-GFP reporter mice depicting endothelial cells (green, middle row) and TRITC70 signal (red, bottom row) 120 min after M2CAO; merged image, top row. TRITC70 extravasation from the vessel lumen into the parenchyma is outlined with dashed lines. TRITC70 is not visible in the green channel, yet the Claudin5-GFP signal can leak into the red channel when GFP expression is very high (asterisks). Scale bars (artery, arteriole, venule, vein) 25 µm, scale bars (capillary) 10 µm. [file 12987_2024_537_MOESM4_ESM.tif]

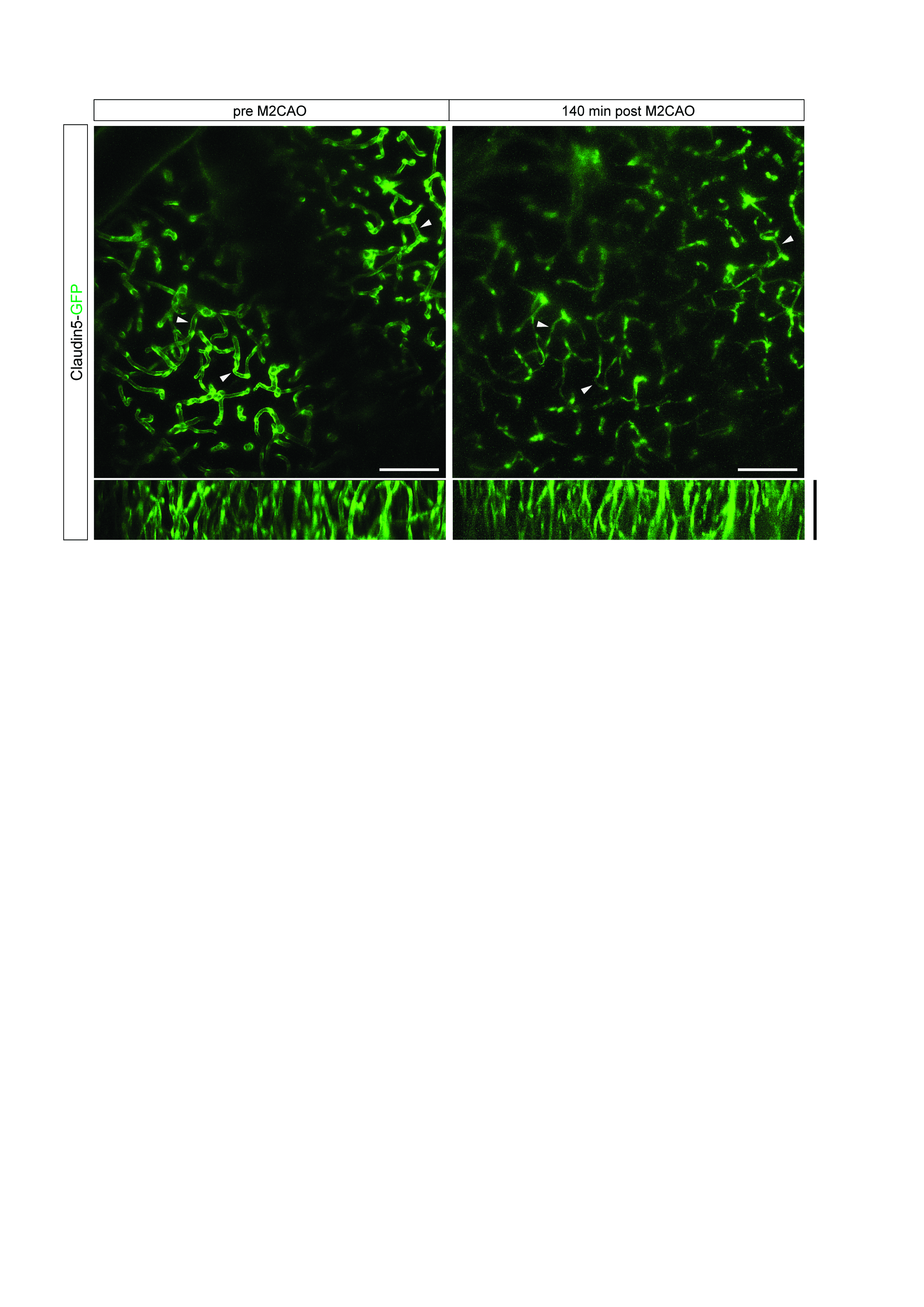

Supplement: Supplementary file 5 — Additional file 5: Figure S4. Vessel constriction within the capillary bed. Maximum intensity and side projections of the capillary bed before and 140 min after M2CAO. Global constriction of the capillaries can be observed (indicated with arrowheads at clearly identifiable vessel segments). Scale bars 100 µm. [file 12987_2024_537_MOESM5_ESM.tif]
